# Hyperactivity in Anorexia Nervosa: Warming Up Not Just Burning-Off Calories

**DOI:** 10.1371/journal.pone.0041851

**Published:** 2012-07-27

**Authors:** Olaia Carrera, Roger A. H. Adan, Emilio Gutierrez, Unna N. Danner, Hans W. Hoek, Annemarie A. van Elburg, Martien J. H. Kas

**Affiliations:** 1 Department of Neuroscience & Pharmacology, Rudolf Magnus Institute of Neuroscience, University Medical Center Utrecht, Utrecht, The Netherlands; 2 Altrecht Eating Disorders Rintveld, Altrecht Mental Health Institute, Zeist, The Netherlands; 3 Departamento de Psicologia Clinica y Psicobiologia, y Unidad Venres Clinicos Facultad de Psicologia, Universidad de Santiago de Compostela, Campus Vida, Santiago de Compostela, Spain; 4 Complejo Hospitalario Universitario de Santiago de Compostela, Santiago de Compostela, Spain; 5 Utrecht Research Group Eating Disorders, Utrecht, The Netherlands; 6 Parnassia Bavo Psychiatric Institute, The Hague, The Netherlands; 7 Department of Psychiatry, Groningen University Medical Center, Groningen, The Netherlands; 8 Department of Epidemiology, Columbia University, New York, New York, United States of America; University of Western Brittany, France

## Abstract

Excessive physical activity is a common feature in Anorexia Nervosa (AN) that interferes with the recovery process. Animal models have demonstrated that ambient temperature modulates physical activity in semi-starved animals. The aim of the present study was to assess the effect of ambient temperature on physical activity in AN patients in the acute phase of the illness. Thirty-seven patients with AN wore an accelerometer to measure physical activity within the first week of contacting a specialized eating disorder center. Standardized measures of anxiety, depression and eating disorder psychopathology were assessed. Corresponding daily values for ambient temperature were obtained from local meteorological stations. Ambient temperature was negatively correlated with physical activity (p = −.405) and was the only variable that accounted for a significant portion of the variance in physical activity (p = .034). Consistent with recent research with an analogous animal model of the disorder, our findings suggest that ambient temperature is a critical factor contributing to the expression of excessive physical activity levels in AN. Keeping patients warm may prove to be a beneficial treatment option for this symptom.

## Introduction

Anorexia nervosa is a mental disorder with high lifetime mortality predominantly affecting adolescent girls and young adult women [1], [2]. Excessive physical activity has been recognized as a paradoxical feature commonly present in AN since the modern description of the disorder by Gull [3] (1874). Up to 40–80% of AN patients show excessive levels of activity referred to as hyperactivity, over-activity, motor restlessness or diffuse restlessness [4], [5]. During treatment this behavior is difficult to control and jeopardizes weight recovery, a central target in the treatment of AN [6].

Although the precise origin and nature of hyperactivity is yet to be ascertained there are several hypotheses trying to explain the role of this behavior in AN. One of these hypotheses retains the historical and widespread view that excessive exercise is linked to the relentless pursuit of thinness [7] and consequently hyperactivity can be considered a deliberate calorie-burning weight reduction strategy under the voluntary control of AN patients. Indeed, the DSM-IV-TR [8] posits hyperactivity as a second order symptom for the diagnosis of AN. Nevertheless, this minor role of hyperactivity and the extent to which patients voluntarily exercise are being strongly questioned as hyperactivity has been shown to play a fundamental role in the development and maintenance of the disorder, in many cases preceding food restriction [9]–[11] and accelerating body weight loss once food restriction has taken place [5].

A second potential explanation considers the role of hyperactivity in AN in term of its anxiolytic and affect regulation properties [12]–[14]. Elevated levels of anxiety and depression are common in compulsively exercising AN patients [15]. In these patients, exercise might be a coping strategy to compensate, suppress or alleviate negative emotional states [16].

Alternatively, in the last decade hyperactivity has been traced to biological needs related to food restriction and weight loss [4], [17]–[19]. This perspective has been substantiated by research on analogous animal models in which rats are submitted to a restricted feeding schedule while having access to a running wheel. Self-starvation and weight loss following from increased running by rats and mice submitted to a restricted feeding schedule in the Activity Based Anorexia model (ABA) [20], [21], [22], [23] revealed a striking parallelism with the symptoms of AN. Besides its interpretation as a form of foraging behavior or its rewarding properties through the activation of dopaminergic reinforcing pathways [5], excessive activity in semi-starved rats has been associated with low levels of the fat-derived hormone leptin [24], association that has also been reported for AN patients [25], [26]. Leptin treatment has been quite effective in suppressing semi-starvation-induced hyperactivity in a modified ABA procedure [27]. In addition, leptin treatment also decreased food intake and increased energy expenditure thermogenesis leading to a worsening of the physical state of already underweight animals [24].

A further hypothesis, coming from ABA research, considers excessive running as a form of thermoregulatory behavior [28]. According to this view rats would increase running in response to hypothermia derived from the restricted feeding schedule and subsequent weight loss. Supporting this hypothesis, ABA research has shown that having access to a warm plate reduced hyperactivity and body weight loss in rats [29]. More importantly, when running had become excessive (and rats had already lost a 20% of their initial body weight) increasing ambient temperature (AT) reversed excessive activity resulting in body weight recovery [30], [31]. Furthermore, preliminary observations suggest that keeping patients warm might reduce physical activity levels [32].

To our knowledge, only one study has explored the association between physical activity and seasonality in AN [33]. However, this article did not report values for AT, and the study was conducted with outpatients recovering from AN (mean BMI = 19.4). These authors found a normalization of increased physical activity in recovering AN patients, as well as a similar seasonal pattern to that displayed by the control group that is, spending more time in low-moderate intensity activity in summer compared with winter time. Thus, the main purpose of the present study was to assess the relative importance of AT, anxiety, depression, and disordered eating attitudes on physical activity in untreated adolescent AN patients. We hypothesized that AT would modulate physical activity levels in these patients.

## Methods

### Participants

An initial sample of 51 consecutive patients at a specialized center for eating disorders in The Netherlands participated in the study. Inclusion criterion for this study was the presence of AN according to the DSM-IV criteria using the Eating Disorder Examination [34] as ascertained by eating disorders experts (all medical doctors), and being female between 12 and 18 years old. Three patients who did not fulfill the weight criterion, but whose weight was clearly below expected from their own growth curves were diagnosed as Eating Disorders Not Otherwise Specified and were also included in the study. Upon confirmation of the eating disorder diagnosis and after obtaining written informed consent form the participants, and/or their parents, three consecutive days of physical activity and additional psychological and anthropometric measures were assessed. All procedures were approved by the Medical Ethics Committee of University Medical Center Utrecht, NL. The final sample was reduced to 37 patients, since three patients did not fill in the initial assessment and 11 patients were discarded due to missing data for objectively measured physical activity (three cases of Actiwatch malfunctioning; two patients did not wear the Actiwatch, and six patients showed long periods of inactivity indicating Actiwatch misuse). According to the mean AT recorded during the three-day period of physical activity, the patients were divided in two groups (Warm vs. Cold). The mean daily outdoor AT was obtained from the Royal Netherlands Meteorological Institute (KNMI; http://www.knmi.nl/klimatologie/daggegevens/index.cgi) for the weather station closest to each patient’s home. All patients except five were outpatients. For inpatients AT data was obtained from the closest weather station to the eating disorder center. Six different weather stations were consulted, mean distance (SEM) from patient’s home 13.73 (1.49) km. According to the mean AT during activity recording (9.2°C), patients assessed under AT conditions of >9.2°C were classified as Warm group (16°C; n = 15 patients) while the remaining 22 were classified as Cold group (4.5°C).

### Measures

#### Objective assessment of physical activity

Physical activity was measured using an accelerometer (Actiwatch model AW 4; Cambridge Neurotechnology, Cambridge, United Kingdom). The Actiwatch was strapped to the patient’s right ankle and worn for three consecutive weekdays, from 9 pm on the first day to 9 pm on the fourth day, except while swimming and showering. The epoch length (sampling time) for the Actiwatch was set to 1 minute. Night activity (23:00–07:00) and sequences of >10 min of consecutive zero counts were excluded from the recordings. This procedure was similar to that recently used in the field of eating disorders [12] and also in children and adult studies [35]. Thereafter, the data were summarized as counts per day and patients were excluded from analyses if more than 30% of the day was not available for 2 of the 3 days. Activity data from days 1 to 3 were averaged to determine daily physical activity for each patient. Data analysis was undertaken to determine the periods of time (%) at varying intensity levels of physical activity. The ranges (in counts per minute) for the activity intensities were <200 for Sedentary activity, 200 to <1800 for Light activity, and ≥1800 for the Moderate to Vigorous (MV) activity, as validated by Puyau et al. [36] for Actiwatch device worn on the lower right leg.

#### Anthropometric measures

The degree of patient underweight was calculated using the body mass index (BMI, Kg/m^2^) computed into Z-scores describing the statistical distance from the mean BMI for that age. Using a software program provided by the Netherlands Organization for Applied Scientific Research TNO, the data were related to Dutch population references [37].

#### Psychological measures

Psychological measures included the following self report instruments: the Eating Disorder Inventory-2 (EDI-2) [38], only the total score and the subscales Drive for Thinness (DT) and Body Dissatisfaction (BD) are presented; the State-Trait Anxiety Inventory (STAI) [39]; and the Children’s Depression Inventory (CDI) [40].

#### Data analysis

Kolmogorov-Smirnov test was used to test normality. Independent t-tests were used to compare Warm and Cold groups. Mann Whitney tests were used if data were not normally distributed (% of time spent at MV activity). A chi square analysis was used to compare Warm and Cold groups in terms of diagnosis subtype and number of inpatients.

A repeated measures ANOVA was performed for the variable counts per hour, with warm and cold conditions as independent factors, and with repeated measures over the daily hours (7:00 to 23:00). In order to further test the effect of AT on activity, a subsample of patients (n = 8) that experienced an AT difference of >4°C for two days of assessment was analyzed separately using a paired t-test to compare within patient activity levels at lower vs. higher AT. Finally, the Pearson’s correlation test was used to explore the association between the following variables: physical activity (counts/day), % of time spent at different activity levels: Sedentary and Light activity, Age, BMI (z scores), AT, STAI-S, STAI-T, CDI and EDI-2. Because DT, BD and % of time spent at MV activity were non-parametrically distributed, the Spearman correlation test was used to explore the association between these and the above mentioned variables. Also, a multiple regression analysis was conducted with physical activity (counts/day) as the dependent variable and AT, BMI (z scores) and STAI-S as predictor variables.

## Results

As shown in Table 1, the AT during the three-day period in which patient wore the Actiwatch was significantly different for Warm and Cold groups, t(35)  = 9.669, p<0.001. Patients’ enrollment in the study for the Warm group was between April and October, while patient referrals to the eating disorders clinic for the Cold group were between October and April. A strong association was observed between the Warm and Cold groups and the standard climatic warm and cold seasons (Spring-Summer and Autumn-Winter, respectively) that correctly classified 90% of patients of the present study. Patients from the Warm group tend to be slightly younger, t(35)  = 2.429, p = 0.02, and less underweight, t(35)  = 2.216, p = 0.033, than those of the Cold group, but no differences were observed between these groups in terms of diagnosis subtype, illness duration or number of inpatients.

**Table 1:** Demographic data for the entire group and, separately, for the Warm and Cold groups.

	Warm group (n = 15)	Cold group (n = 22)	Total (n = 37)
Age, mean (range; SD) years	14.7 (13–17; 1.34)*	15.67 (13–17.5; 1.04)	15.3 (1.25)
BMI, mean (SD) kg/m2	16.38 (1.25)	15.75 (1.65)	16 (1.5)
BMI, mean (SD) Z scores	−1.83 (0.75)*	−2.74 (1.5)	−2.38 (1.31)
Duration of illness, mean (SD) years	1.11 (0.66)	1.26 (0.89)	1.2 (0.79)
ANR, n (%)	10 (67)	15 (68)	25 (68)
ANP, n (%)	4 (27)	5 (23)	9 (24)
EDNOS, n (%)	1 (7)	2 (9)	3 (8)
Inpatients, n (%)	2 (13)	3 (14)	5 (13)
Ambient Temperature, mean (SD) °C	16 (4.21)**	4.5 (3)	9.2 (6.79)

ANR: Anorexia Nervosa Restricting type; ANP: Anorexia Nervosa Binge Eating/Purging type; EDNOS: Eating Disorders Not Otherwise Specified. Significance differences between Warm and Cold groups, *p<.05; **p<.001.

**Figure 1:**
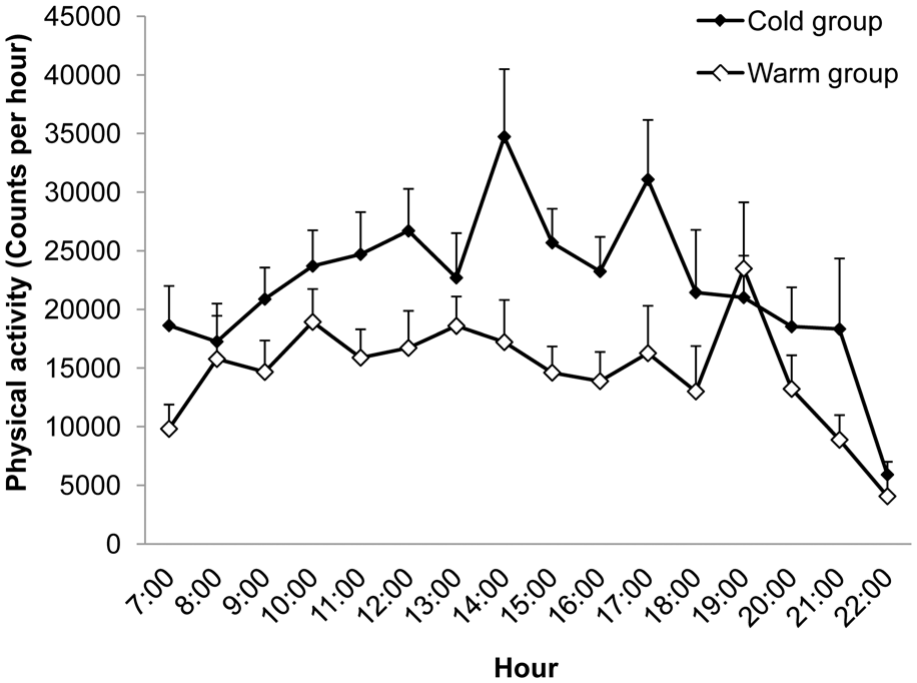
Physical activity over daily hours for the Warm and Cold groups. Mean (SEM) physical activity over daily hours (counts/hour) for the Warm and Cold groups (3-days measurement). Overall activity was higher for the Cold group, p<.01.

Table 2 shows that the Cold group was significantly more physically active (counts/day) than the Warm group, p = 0.003. Also, the Cold group spent more time at the Light and MV activity levels, p = 0.022 and p = 0.020 respectively. Consistently with these findings, the Warm group spent more time at the Sedentary levels of activity, p = 0.004. No further significant differences were observed for the remaining psychological measurements between the Warm and Cold groups, as shown in Table 2. The pattern of physical activity for the Warm and Cold groups over daily hours (from 7:00 to 23:00) is shown in Figure 1, with higher levels of physical activity for the Cold group. A repeated measures ANOVA (controlling for BMI z scores) showed a significant main effect for the Warm vs. Cold condition, F (1, 34)  = 6.641, p = 0.014, confirming that overall activity was significantly increased for the Cold group. Time (daily hours) by group interaction was not significant.

**Table 2:** Physical activity, time spent per day in various levels of physical activity and psychological measures, mean (SD), for the Warm and Cold groups.

	Warm group (n = 15)	Cold group (n = 22)		Cohen’s d
Physical activity (counts/day)	230067.9 (77421.33)	347266.75 (128808.38)	t(35) = 3.149**	1.1
S. Activity (%)	72.08 (5.42)	64.38 (6.85)	t(35) = 3.085**	1.24
Lig. Activity (%)	24.14 (2.01)	29.18 (2.65)	t(35) = 2.393*	2.14
MV activity (%)	3.78 (2.24)	6.47 (3.47)	U = 90.000*	0.92
STAI-S	52.6 (13.54)	52.36 (12.95)	t(35) = 0.054	0.02
STAI-T	56.80 (11.31)	58.18 (12.36)	t(35) = 0.346	0.12
CDI	19.93 (7.15)	21 (9.83)	t(34) = 0.357	0.12
EDI-2	317.67 (54.66)	318.98 (54.94)	t(35) = 0.070	0.02
DT	34.02 (8.08)	34.84 (7.24)	U = 160.500	0.12
BD	43.07 (10.29)	42 (10.22)	U = 152.500	0.10

STAI-S: S. Activity: Sedentary activity; Lig. Activity: Light activity; MV activity: moderate to vigorous activity; State anxiety; STAI-T: Trait anxiety; CDI: Children Depression Inventory; EDI-2: Eating Disorders Inventory-2 (total score); DT: Drive for Thinness; BD: Body Dissatisfaction. Cut-offs for Sedentary, Light and MV physical activity were <200, 200–1800 and >1800 counts/min respectively. *p<.05; **p<.01.

Irrespective of group, correlation analyses have been performed for the whole sample to explore other factors that could be contributing to the expression of hyperactivity in AN. As shown in Table 3, AT was the variable showing the strongest correlation with physical activity. Also, a negative correlation was detected for MV physical activity and STAI-S, that is, the more time spent in MV physical activity the less anxiety reported. A multiple regression analysis was conducted with physical activity (counts/day) as the dependent variable and AT, BMI (z scores) and STAI-S as predictor variables. The entire model explained 18% of the variance on physical activity scores, Adjusted R^2^ = 0.18; F (3, 36) = 3.690, p = 0.021. Only AT accounted for a significant portion of the variance in physical activity (counts/day), β = −0.358; t = −2.210, p = 0.034. Other variables (STAI-T, CDI, EDI-2, and AGE in months) were also entered as predictor variables, but none of these different predictors entered were significant, all p>0.05.

**Table 3:** Cross sectional correlations between physical activity levels, BMI (z scores) and psychological measurements for the whole sample (n = 37).

	AGE	BMI	PA	S. PA	Lig. PA	MVPA	AT	STAI-S	STAI-T	CDI	EDI-2	DT	BD
AGE	–	−310	.126	−.184	.167	.112	−.392*	.160	.071	.171	−.089	.252	.263
BMI		–	−.314	.147	−.034	−.118	−.358*	−.172	−.051	−.334*	−.225	.036	−.045
PA			–	–	–	–	−.405*	−.147	.001	.088	.036	−.074	−.141
S. PA				–	–	–	.427**	.197	−.012	.016	.116	.065	.159
Lig. PA					–	–	−.344*	−.143	.080	.002	−.145	−.028	−.153
MVPA						–	−.406*	−.348*	−.212	−.134	−.128	−.118	−.088
AT							–	−.145	−.189	−.122	−.089	−.025	−.035
STAI-S								–	.680**	.738**	.572**	.490**	.597**
STAI-T									–	.631**	.565**	.519**	.612**
CDI										–	.701**	.544**	.504**
EDI-2											–	.719**	.697**
DT												–	.757**
BD													–

BMI: BMI (z scores); PA: Physical activity (counts/day); S. PA: Sedentary physical activity; Lig. PA: Light physical activity; MVPA: Moderate to vigorous physical activity; AT: Ambient temperature; STAI-S: State-Trait Anxiety Inventory-State; STAI-T: State-Trait Anxiety Inventory-Trait; CDI: Children Depression Inventory; EDI-2: Eating Disorders Inventory-2 (total score); DT: Drive for Thinness; BD: Body Dissatisfaction. Correlations for the different activity measures are not shown because they are related measures. *p<.05; **p<.01.

**Figure 2:**
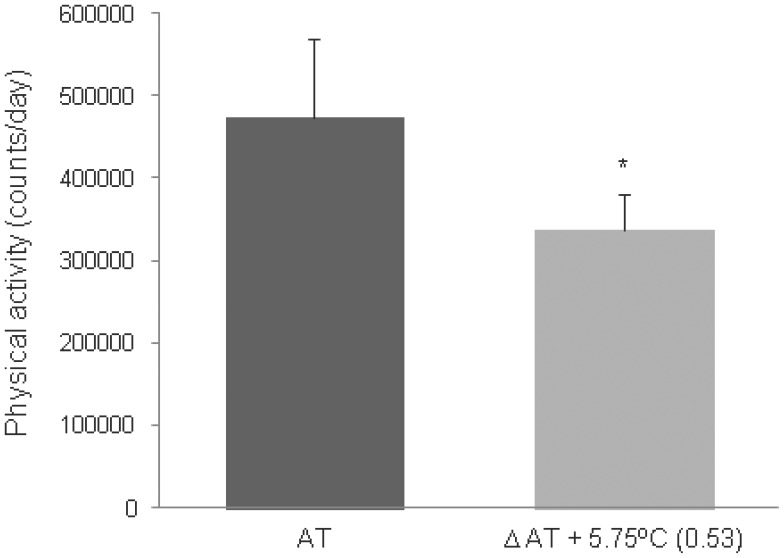
Differences in physical activity as a function of AT for a subsample of 8 patients. Mean (SEM) physical activity levels (counts/day) as a function of AT for a subsample of 8 patients that showed a >4°C difference of AT over consecutive days. AT =  lowest mean AT; Δ AT =  mean difference between the lowest and highest AT. Patients were more active at lower AT, *p<.05. Cohen’s d = 0.89.

In order to further clarify the effect of AT apart from length of day or other seasonality confounding effects, a paired samples t-test (one-tailed) was conducted for a subsample of 8 patients that experienced a variation of AT >4°C (mean (SD), 5.76 (1.41) between two consecutive days in the three-day period of physical activity assessment. As shown in Figure 2, patients displayed higher levels of Physical activity on the lower AT day, t (7) = 2.123, p = 0.035, confirming the robust effect of AT on activity.

## Discussion

The main finding of this study was the significant effect of AT on physical activity in adolescent AN patients i.e., patients express higher activity levels during the cold months. This association between AT and physical activity in AN also contrasts with data for the general population and other disorders, such as obesity, where higher levels of physical activity have been reported in the warmer months than in colder months [41], [42]. Furthermore, this association seems to exclude the mediation of other climatic aspects, as length of day or seasonality that are associated to AT, as shown by the result in the subsample of patients experiencing an AT difference >4°C between two consecutive days during the three days period of activity recording. Despite the fact that physical activity for these patients was significantly higher at the lowest temperature and psychopathological assessment (depression, anxiety) was performed in the same week as activity monitoring, these differences in activity did not rule out the potential mediation of day-to-day changes on psychological well-being or any other situational shifts of daily physical activity.

With respect to the potential role of increased physical activity to cope with anxiety [25], or in the regulation of negative affect [12] we did not identified anxiety and depression as relevant predictor variables of physical activity levels in the multiple regression analysis, nor were there significant differences on these variables between the Warm and Cold groups. Based on the factors tested, the multiple regression analysis showed that AT was the only significant predictor of physical activity. Furthermore, the modulation of physical activity in consecutive days detected in the subgroup of patients experiencing a variation of AT of >4°C calls for more attention to be paid to AT in future research studying the affective-emotional regulation role of excessive activity in AN, in order to determine the relative contribution of both factors (AT and affect regulation) to the expression of physical activity.

Our results seem to support the thermoregulatory hypothesis used to explain the running behavior in semi-starved rats [31]. According to this hypothesis, the increase in physical activity observed in AN patients during the colder months of the year may be an adaptive response to compensate for the hypothermia derived from defective insulation due to body weight loss. Hypothermia is a common sign in AN patients who frequently complain of feeling cold and many of them warm themselves by having hot drinks, sitting close to heaters, and covering themselves with warming blankets [43]. Thus, according to our results, physical activity in untreated AN patients could be more to the service of warming them up than a conscious strategy to burn off calories. In view of this, it is worth noting that subscales from the EDI-2 measuring drive for thinness and body dissatisfaction were not associated with any objective measure of physical activity. In addition, it has been recently reported that eating disorder patients rate exercise for fitness-related reasons as less important than controls, whereas no differences emerged regarding exercise for weight/appearance related reasons [12]. These findings challenge the commonly held belief that physical activity is just a mere weight-losing strategy deliberately employed by AN patients, and underscores the need for reappraising the role of hyperactivity in AN as a key biologically driven feature of the disorder.

Moreover, the association between AT and hyperactivity is coherent with the biased distribution of AN incidence across latitudes, one of the main factors determining AT [44]. In comparison with the small number of reports from tropical countries [45], most of the epidemiological studies conducted up to date have been performed in populations located between 40° and 65° latitude in the northern hemisphere, a latitude band that closely corresponds to the Temperate climates zones in the Köppen-Geiger climate classification [46]. Of note, two thirds of the literature references have been conducted in this latitude band, as it happened for Psoriasis, a medical disorder known to be bounded to higher latitudes [44]. This study does not exclude culture-bound hypothesis but provides a complementary perspective of the term environment by encompassing other factors that have been overlooked and may influence AN. Additional evidence regarding the effect of AT on AN is the recent report of the favorable influence of warm seasons in menses resumption in AN patients, in spite of the fact that patients body weight was on average 2 kg less during warmer seasons [47].

One limitation of this study is the lack of data concerning patient’s indoor temperature, and the possible use of different warming strategies (e.g., drinking hot liquids). However, in epidemiological research outdoor AT has been extensively used as a surrogate for personal exposure to heat and cold, as AT is the strongest determinant of variation over time in the exposure of populations to high and low ATs [48]. Another limitation of the study is the absence of data from healthy controls. However, earlier studies have shown that physical activity levels in the normal population and in disorders such as obesity, is related to AT in an opposite way than we found here for AN [41], [42]. A further limitation of the study is the absence of leptin data which prevented us to determine the role of leptin levels on physical activity and therefore to rule out the relative contribution of leptin and AT on physical activity. Furthermore, the final sample size of this study was 37 patients. Therefore, this is an initial study and replication of these findings in view of larger sample sizes would be necessary to further support the role of AT in the expression of excessive physical activity in AN.

In summary, the association detected between AT and physical activity offers a fresh perspective on our understanding of the disorder and opens new avenues for the management of excessive physical activity, one of the AN symptoms most refractory to treatment. In line with the heat reversal of excessive running and body weight loss exhibited by animals exposed to the analogous ABA model [31], decreased physical activity may be the unrecognized effect underpinning the first recommendation in the literature of supplying patients with external heat as suggested by Gull [3] (1874) for the treatment of AN [32], [49].
